# Centering the marginalized: AI-driven strategies for advancing health equity in rare disease care

**DOI:** 10.1016/j.patter.2026.101535

**Published:** 2026-05-08

**Authors:** Chaoyu Lei, Ying Zuo, Claudia Abreu Lopes, Jaimee Stuart, Benjamin Xu, T.Y. Alvin Liu, Thomas Ploug, Zilong Wang, Kang Dang, Kai Jin, Haoxuan Yu, Heaven Yeshaneh Tatere, Fanyi Kong, Ning Zhang, Lufa Zhang, Huifang Zhou

**Affiliations:** 1 State Key Laboratory of Eye Health, Department of Ophthalmology, Shanghai Ninth People’s Hospital, Shanghai Jiao Tong University School of Medicine, Shanghai, China; 2 Hainan International Medical Center, Shanghai Jiao Tong University School of Medicine, Hainan, China; 3 School of International and Public Affairs, Shanghai Jiao Tong University, Shanghai, China; 4 United Nations University International Institute for Global Health, Kuala Lumpur, Malaysia; 5 United Nations University Institute in Macau, Macau SAR, China; 6 Keck School of Medicine, Roski Eye Institute, University of Southern California, Los Angeles, CA, USA; 7 Wilmer Eye Institute, Johns Hopkins University School of Medicine, Baltimore, MD, USA; 8 Centre for AI Ethics, Law, and Policy, Department of Communication and Psychology, Aalborg University, Copenhagen, Denmark; 9 School of Health and Welfare, Halmstad University, Halmstad, Sweden; 10 Microsoft Research Asia, Shanghai, China; 11 School of AI and Advanced Computing, XJTLU Entrepreneur College (Taicang), Xi’an Jiaotong-Liverpool University, Suzhou, Jiangsu, China; 12 Eye Center of Second Affiliated Hospital, School of Medicine, Zhejiang University, Hangzhou, Zhejiang, China; 13 Zhejiang Provincial Key Laboratory of Ophthalmology, Zhejiang Provincial Clinical Research Center for Eye Diseases, Zhejiang Provincial Engineering Institute on Eye Diseases, Hangzhou, Zhejiang, China; 14 UMass Chan Medical School, Worcester, MA, USA; 15 Institute for Hospital Management of Tsinghua University, Shenzhen, Guangdong, China; 16 School of Healthcare Management, Tsinghua University School of Medicine, Beijing, China; 17 Department of Obstetrics and Gynecology, NHC Key Laboratory of Study on Abnormal Gametes and Reproductive Tract, the First Affiliated Hospital of Anhui Medical University, Hefei, Anhui, China; 18 Institute for Urban Governance, Shanghai Jiao Tong University, Shanghai, China; 19 Institute of Healthy Yangtze River Delta, Shanghai Jiao Tong University, Shanghai, China

**Keywords:** rare disease, artificial intelligence, health equity, fairness, ethics

## Abstract

Rare diseases (RDs) affect 6%–8% of the global population but remain critically underserved. People living with an RD face misdiagnosis, limited treatment options, and inequitable access to specialized care. While artificial intelligence (AI) offers transformative potential in RD care, significant challenges remain. This perspective identifies five key dimensions to equitable AI application in RD care: data availability, algorithmic fairness, patient privacy, resource prioritization, and medical ethics. To address these barriers, strategies include enhancing data diversity through internationally harmonized repositories, leveraging synthetic data, and employing fairness-aware algorithms. Privacy-preserving methods safeguard sensitive genetic data while enabling collaborative research. Transparent resource-allocation frameworks and interdisciplinary governance ensure equitable distribution of AI-driven benefits, particularly in low- and middle-income countries. Ethical considerations, including patient-centered consent and dynamic risk assessments, are foundational to sustainable AI integration. By addressing these multidisciplinary challenges, AI can advance health equity, transforming RD care from fragmented and inequitable to inclusive and innovative. This paradigm shift aligns technological progress with the ethical imperative to ensure no patient is left behind in the promise of precision medicine.

## The bigger picture

Rare diseases, though individually uncommon, collectively impact hundreds of millions worldwide, often leading to prolonged diagnostic journeys, limited treatment choices, and uneven access to expert care, especially in underserved communities. Artificial intelligence (AI) is emerging as a powerful tool in medicine, capable of analyzing vast amounts of data to identify patterns, predict outcomes, and personalize treatments that could revolutionize care for these overlooked illnesses. Without careful design, however, AI risks widening existing gaps, for example, when training data overlook diverse populations or when privacy concerns hinder global collaboration, potentially leaving low- and middle-income countries or ethnic minorities further behind. By prioritizing fair algorithms, secure data sharing, and ethical guidelines that prioritize patient voices, AI can help drive inclusive progress, turning rare disease management from a patchwork of inequalities into a model of equitable precision medicine. This approach could not only improve outcomes for those affected but also strengthen overall health systems, demonstrating how technology, when aligned with social justice, can foster a more compassionate and effective global medical landscape.

## Teaser

Rare diseases impact 6%–8% of the global population, yet patients face frequent misdiagnosis, scarce treatments, and unequal access to medical care. This perspective shows that AI could transform rare disease care but only if designed to promote equity rather than deepen disparities. It identifies five core dimensions (data availability, algorithmic fairness, patient privacy, resource prioritization, and medical ethics) and recommends practical strategies. These steps can make AI a powerful force for health equity, ensuring precision medicine truly includes everyone.

## Introduction

Rare disease (RD) is defined by low prevalence, although thresholds vary across jurisdictions. In the European Union (EU), an RD affects fewer than 1 in 2,000 individuals, whereas in the United States (US), it is defined by an absolute threshold of fewer than 200,000 affected individuals.^1^ Despite these definitional differences, more than 7,000 RDs collectively affect an estimated 6%–8% of the global population, corresponding to approximately 262.9–446.2 million people worldwide.^2^^,^^3^ In the US alone, the annual economic burden of RD approaches $1 trillion, excluding the substantial and enduring socioeconomic impact on patients, families, and caregivers.^2^

Despite the substantial burden of disease in this group, RD remains critically underserved by healthcare innovation due to limited clinical evidence, disease recognition complexity, and high costs of developing treatments.^4^ People living with an RD (PLWRDs) face misdiagnosis or prolonged diagnostic delays, scarce treatment options, and limited access to specialized expertise to meet their needs.^5^ This “marginalized majority” paradox (collectively affecting hundreds of millions yet individually overlooked) underscores the urgent need to ethically recalibrate global health priorities to ensure equity for RD communities, defined as the fair distribution of healthcare resources and opportunities that allow all individuals to achieve comparable health outcomes regardless of disease rarity.

**Figure 1:**
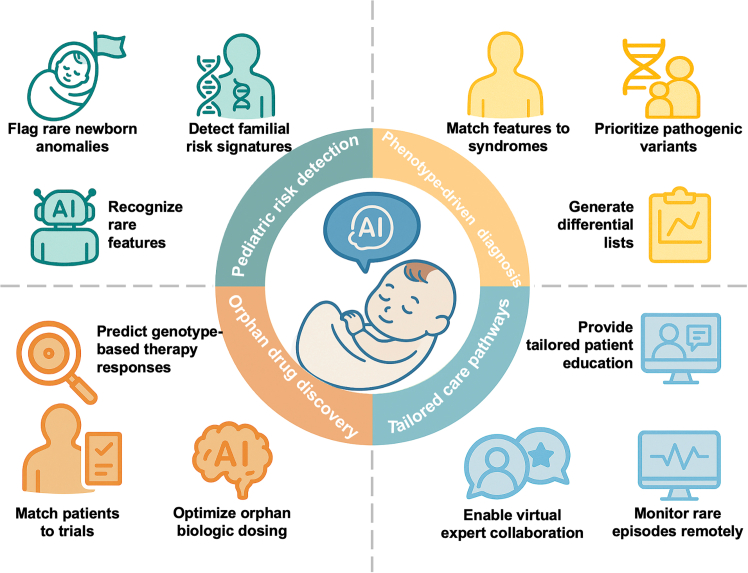
AI applications in rare disease care Four aspects: pediatric risk detection, phenotype-driven diagnosis, orphan drug discovery, and tailored care pathways.

At the 2025 World Health Assembly, the World Health Organization (WHO) spotlighted RDs by exploring technological innovations, including artificial intelligence (AI), to enhance access to specialists and treatments.^6^ AI refers to technologies that enable machines to perform tasks requiring human-like intelligence. It includes machine learning (ML), with deep learning (DL) dominating current medical applications.^7^ These innovations are transforming RD care by detecting subtle patterns in complex data, such as genomics and medical imaging, while also reducing diagnostic development costs and easing reliance on limited clinical expertise (Figure 1).^8^^,^^9^^,^^10^ For example, a DL-based point-of-care screening tool using facial images was developed to detect 128 genetic syndromes in children.^11^ Recent advances of foundation models further demonstrate their emerging promise in RD care. For example, MedFound, a 176-billion-parameter language model, achieved 80.7% top-3 diagnostic accuracy across 2,105 RDs in zero-shot evaluation settings.^12^

However, RD is structurally distinguished from common conditions in ways that fundamentally reshape the ethical, technical, and policy landscape for AI deployment. These distinctions include an extreme long-tail data distribution, amplified privacy risks arising from small and highly identifiable patient populations, systemic deprioritization driven by low prevalence and limited return on investment, and the resulting marginalized majority paradox (Table 1). Together, these features create AI equity risks that differ qualitatively from those encountered in common diseases. The WHO’s Global Initiative on Artificial Intelligence for Health (GI-AI4H) has underscored the urgency of prioritizing underrepresented groups, including PLWRDs.^13^ To ensure that AI-driven innovations advance health equity and benefit all patients, this perspective aims to address these challenges from a multidisciplinary clinical, technical, and ethical perspective.

**Table 1:** Structural differences in AI equity challenges: Common versus rare diseases

Dimension	Common diseases	Rare diseases	Equity implications for AI in rare diseases
Data availability	(1) abundant datasets from diverse sources (2) established multi-source registries	(1) extreme scarcity and fragmentation across sites (2) reliance on synthetic data and patient-powered registries	models overlooking or excluding ultra-rare conditions; synthetic data risks diluting rare signals
Algorithmic fairness	(1) addressable moderate class imbalance (2) more homogeneous phenotypes within categories	(1) long-tail distribution and architecture-induced bias (2) profound intra- and inter-disease variability	requiring highly tailored models; generalization failures more consequential; biases amplified in underrepresented classes
Patient privacy	(1) lower re-identification risk due to large sample sizes (2) anonymization can protect identities	(1) genetic uniqueness enables re-identification from minimal data (2) demands cryptographic methods	standard privacy methods insufficient; collaboration hindered by heightened risks
Resource prioritization	(1) high ROI drives investment in AI solutions (2) cost-effectiveness analyses favor population-level impact	(1) non-viable ROI for bespoke AI models (2) systemic underfunding	systemic underfunding of RD-specific AI; zero-sum trade-offs intensified
Medical ethics	(1) shorter diagnostic journeys, broader expertise (2) advocacy aligns with disease prevalence	(1) prolonged odyssey, high lifetime costs (2) LMICs face competing priorities	equity gaps amplified globally (especially in LMICs); AI should prioritize unmet needs and societal burden over average performance

RD, rare disease; ROI, return on investment; LMICs, low- and middle-income countries.

## Challenges and strategies for applying AI to RD care

This perspective presents a conceptual and normative framework to ensure that AI contributes to advancing RD care ethically and equitably. This framework is derived from a synthesis of the published literature, illustrative real-world case examples, and the authors’ collective interdisciplinary experience across clinical, technical, ethical, and policy domains. It is intended as an orientation and decision-support framework rather than a formally derived consensus or empirical model.

**Figure 2:**
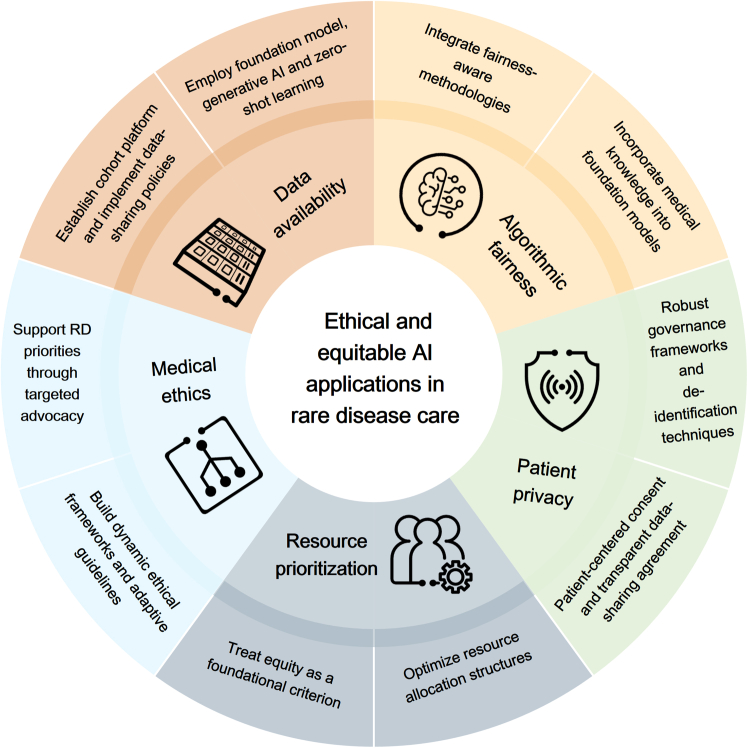
Strategies for applying AI in rare disease care ethically and equitably Five dimensions: data availability, algorithmic fairness, patient privacy, resource prioritization, and medical ethics.

Organized around the RD-specific structural differences outlined in Table 1, the framework comprises five key dimensions that should be considered in equitable application of AI in RD care: (1) data availability, (2) algorithmic fairness, (3) patient privacy, (4) resource prioritization, and (5) medical ethics. As illustrated in Figure 2, each dimension poses specific challenges and corresponding mitigation strategies. In the following sections, each dimension is discussed in the context of RD-specific realities. Technical terms for each dimension are summarized in Table 2.

**Table 2:** Summary of technical terms in AI for RD

Dimension	Term	Definition	Significance in RD context
Data availability	foundation model	large-scale AI models pre-trained on broad, often multimodal datasets	enables generalization capabilities for RD
	generative model	AI architectures that create synthetic data mimicking real-world distributions	compensates for RD data scarcity by simulating data for model training
	generative adversarial network (GAN)	dueling neural networks (generator versus discriminator) producing synthetic data	generates realistic RD-related data (e.g., facial or imaging phenotypes) when real samples are scarce
	diffusion model	generative AI that creates data by reversing gradual noise addition	produces high-fidelity synthetic medical images for ultra-rare conditions
	zero-shot learning	model diagnoses diseases not seen during training	enables AI diagnosis for RD lacking training data via foundation models
Algorithmic fairness	adversarial debiasing	technique training models to suppress bias from sensitive attributes via adversarial networks	prevents algorithmic neglect of RD subtypes in long-tail distributions
	fairness-aware algorithms	methods to detect/mitigate biases	counters algorithmic neglect of RD by suppressing biased attributes in model decisions
	knowledge-based AI	AI integrating medical domain knowledge into architectures	embeds clinical expertise to interpret multimodal RD data where patterns are sparse
Patient privacy	federated learning	decentralized training: data stay local; only model updates shared	enables global collaboration without transferring sensitive genetic data
	differential privacy	anonymization via statistical noise addition to individual data	protects genetically unique patients with RD from re-identification in small cohorts
Resource prioritization	Pareto optimality	resource state where no reallocation can improve one outcome without harming another	exposes RD equity-efficiency tradeoff: optimizing population health often marginalizes people with RD
Medical ethics	structural discrimination	systemic biases in policies/funding that disadvantage marginalized groups	explains RD neglect in LMICs where public health prioritizes high-mortality diseases

### Data availability

Stemming directly from the extreme long-tail data scarcity that defines RD (Table 1), one of the most fundamental challenges for AI innovation in RD is the limited availability of comprehensive, high-quality, and interoperable data. The clinical and genetic datasets of RDs are often sparse, fragmented, and geographically dispersed.^14^ While registries such as the European Reference Networks (European Platform on Rare Disease registration)^15^ and the NIH/NCATS Global Rare Diseases Patient Registry Data Repository (GRDR)^16^ exist, they frequently fall short of fully incorporating international interoperability standards.^17^ Consequently, AI models trained on such datasets may excel within overrepresented subgroups but fail to generalize across heterogeneous RD populations.

In addition, the diagnostic processes for RD are frequently complex and multimodal, requiring integration of phenotypic, genomic, imaging, and longitudinal clinical data. For instance, AI-driven facial analysis tools, such as DeepGestalt, have achieved a top-10 accuracy of 91% across 502 facial images covering 92 different RD syndromes.^18^ However, this approach remains limited by single-modality training data and narrow coverage of conditions. Inconsistent data collection practices, inadequate infrastructure in low- and middle-income countries (LMICs), and a lack of regulatory frameworks for cross-border health data exchange further exacerbate these challenges.

#### Proposed strategy: Enhancing data collection and diversity

In 2021, the United Nations (UN) adopted the resolution on “Addressing the challenges of PLWRDs and their families,” ratified by 193 member states that agreed on the collection, analysis, and dissemination of data on PLWRDs, disaggregated by income, sex, age, race, ethnicity, migration status, disability, geographical location, and other characteristics.^19^ To reduce the impacts of data scarcity, it is important to aggregate RD data from multiple sources or sites and adopt a common data model (CDM) such as the Observational Medical Outcomes Partnership (OMOP).^20^ Tailoring the OMOP CDM to RDs (RD-CDM) can address interoperability gaps in real clinical and research workflows. For example, an OMOP-based RD-CDM integrated phenotypic and genomic data from over 61,000 patients across 4 clinical domains, enabling multi-center extract-transform-load processes and AI-driven genotype-phenotype analyses.^21^ Additionally, an ontology-based RD-CDM comprising 78 data elements has been implemented in REDCap at four German university hospitals, facilitating consistent real-world data capture, registry submissions, Phenopacket-based analyses, and harmonization with fast healthcare interoperability resources (FHIR) and international standards.^17^ These examples illustrate how RD-CDMs address interoperability not only at the scheme level but within operational clinical and research workflows, transforming fragmented RD data into semantically unified, AI-ready resources.

Establishing a long-term cohort platform specifically for RD populations is vital for developing high-quality biomedical databases. While internationally harmonized data repositories could enable capturing RD data in real time, restrictive national data privacy policies often prohibit cross-border health data exchange. This regulatory fragmentation stifles progress, underscoring the urgent need for international alignment on data-sharing frameworks to unlock the potential of collaborative RD research. Patient-led initiatives such as the “Count Me In” project show how patient-powered registries can accelerate genomic discoveries in rare cancers.^22^

In addition, generative models such as generative adversarial networks (GANs) and diffusion models to generate synthetic data demonstrate emerging potential to address the scarcity of data in RD.^23^^,^^24^ Moreover, foundation models trained on massive datasets may offer generalization capabilities for RD, as exemplified by a pathology foundation model for rare cancer.^25^ Generative foundation models such as MetaGP have also shown initial potential in addressing unmet clinical needs for RD.^26^ Notably, zero-shot learning capabilities in vision-language foundation models (e.g., RetiZero) could enable hypothesis-driven RD detection in settings where task-specific labeled data are unavailable.^27^ However, these approaches carry risks of overconfidence, poor probability calibration, and hidden demographic biases, issues that can result in underdiagnosis of marginalized or underrepresented groups and potentially exacerbate inequities for underserved patients with RD.^28^^,^^29^ Consequently, before clinical deployment, these models should undergo calibration assessment, independent external validation, and subgroup-level performance analysis with uncertainty reporting, to avoid overconfident predictions that may exacerbate inequities.

### Algorithmic fairness

Arising from their position in the extreme long tail of classification tasks and their profound heterogeneity (Table 1), RD presents inherent challenges for AI algorithms that depend on historical data to learn predictive patterns. When training datasets carry hidden biases, the resulting models can perpetuate and even amplify existing disparities.^30^^,^^31^ Existing mitigation strategies, including synthetic data generation and ensemble modeling, can mitigate but not eliminate bias arising from class imbalance. Importantly, many widely adopted loss functions and evaluation metrics optimize population-level performance, which may unintentionally disadvantage RD subgroups by assigning them limited influence during training. Furthermore, oversampling increases overfitting risk in small cohorts, while ensemble-based solutions demand substantially greater computational and modeling resources.^32^

These challenges, combined with uneven representation of disease phenotypes and inconsistent annotations by human experts, skew AI models and hinder their performance when applied to RD. A pertinent example illustrating these challenges is a study on dermatological AI models. Researchers assessed state-of-the-art dermatology AI systems using the Diverse Dermatology Images dataset.^33^ Notably, the results of the research found that the models exhibited substantially decreased accuracy on images of dark skin tones and uncommon diseases.

#### Proposed strategy: Mitigating algorithmic bias and upholding fairness

Integrating fairness-aware methodologies at the outset of AI development is critical to counteract biases embedded in historical data. For example, adversarial debiasing is an ML technique that trains models to make accurate predictions while minimizing influence from attributes such as ethnicity or gender, which may contribute to bias.^34^ Meta-learning enables AI systems to rapidly adapt to new RDs by leveraging knowledge from related tasks, which can reduce reliance on large, disease-specific training datasets and help mitigate algorithmic bias against underrepresented conditions.^35^ These approaches can reduce reliance on spurious correlations that exacerbate health disparities. To ensure equitable outcomes, developers could create RD-specific test sets and fairness metrics, for example, evaluating performance across different age groups or ethnicities. Regular human-centered fairness audits and interdisciplinary collaboration will align AI tools with both technical excellence and equity objectives.

Notably, a paradigm shift is underway toward knowledge-based AI that thrives on small, complex data by incorporating medical domain knowledge. Embedding expert knowledge into foundation models further enhances model performance. This is exemplified by MINIM, a recently developed unified medical image-text generative model.^23^ MINIM generates synthetic medical images depicting diverse organs across multiple imaging modalities based on textual descriptions, effectively augmenting scarce real data.^23^ In addition, causally aware synthetic data frameworks, such as DECAF, that generate fair data from biased data ensure that protected attributes do not drive downstream model prediction while preserving underlying causal relationships.^36^ However, synthetic data are not without practical limitations: generation often overlooks or dilutes clinically significant rare or ultra-rare phenotypes and can amplify biases from dominant patterns in training data (eroding signals essential for equitable representation of long-tail distributions).^29^^,^^37^ Careful validation against real-world utility and diversity preservation are therefore essential before deployment.

### Patient privacy

Amplified by the small population sizes and uniquely identifiable phenotypic-genetic profiles (Table 1), RD research relies heavily on sensitive clinical and genetic information. This makes it particularly vulnerable to privacy breaches and cybersecurity threats that may lead to discrimination against PLWRDs in terms of education, employment, insurance, and healthcare access. It is estimated that a genetic database needs to cover “only 2% of the target population to provide a third-cousin match to nearly any person” in a matching attack.^38^ Even de-identified datasets in RD contexts may be susceptible to re-identification due to their small sample size and uniquely distinguishable phenotypic-genetic profiles.^39^^,^^40^ Advances in computational techniques, such as inference algorithms, have also lowered the barrier for malicious attacks and can uncover personal attributes.

Regulatory frameworks such as the General Data Protection Regulation (2018) and the EU AI Act (first proposed in 2021) provide foundational safeguards, but technology evolves rapidly, often outpacing policy.^9^ Advanced anonymization methods, including differential privacy and machine unlearning, obscure individual contributions by adding statistical noise but require precise noise calibration.^41^ Federated learning enables collaborative model training on distributed datasets.^42^ However, it requires a robust cross-institutional infrastructure, synchronized update protocols, and stringent governance. Moreover, next-generation phenotyping tools that leverage facial images, such as DeepGestalt, pose unique privacy challenges.^18^

#### Proposed strategy: Strengthening data governance and privacy protections

Robust governance frameworks are vital for safeguarding sensitive information in RD research, where datasets often include personal genetic data. Strict protocols for informed consent, de-identification, and granular access controls protect patient privacy while permitting critical research. However, as Paul Ohm notes, “data can be either useful or perfectly anonymous, but never both,” highlighting the inherent trade-off between data utility and privacy.^43^ Thus, advanced de-identification techniques should continually aim to balance between preserving data utility and minimizing re-identification risk.^44^ Recent research indicates that even datasets compliant with privacy regulations may remain vulnerable to re-identification through linkage attacks or membership inference, especially when combined with publicly available information.^45^ The use of synthetic data is also a potential solution to protect patient confidentiality.^46^ However, it remains imperfect in practice, particularly for ultra-RDs, as unique phenotypic-genetic profiles may persist, enabling re-identification risks.^37^ Thus, synthetic data should be regarded as a conditional intervention that requires careful validation, governance, and complementary safeguards rather than a standalone remedy for scarcity and privacy constraints.

Patient-centered consent processes and transparent data-sharing agreements help patients understand how their information will be used and allow them to decide when to consent or to withdraw data sharing. Technology-enabled mechanisms such as smart contracts enable dynamic, auditable, and patient-centered consent.^47^ Such approaches may allow data use to align more closely with individual preferences and enhance both transparency and trust.^47^ Regulatory bodies are encouraged to adopt agile standards, conducting periodic policy reviews and issuing guidelines to address emerging risks such as synthetic data misuse. Embedding privacy as a foundational principle is essential to sustaining public trust and ethical integrity in AI-driven care. In addition, a federated governance model, exemplified by the American Thrombosis and Hemostasis Network (ATHN), demonstrates how secure aggregation of clinical and genetic information across treatment centers can comply with HIPAA regulations.^48^

### Resource prioritization

Driven by the low prevalence and consequent low return on investment that systematically marginalizes patients with RD (Table 1), healthcare systems and AI innovations have historically prioritized high-prevalence diseases due to their better population-level outcomes and higher economic benefits. If optimization for cost-effectiveness is framed as a zero-sum game, then it may fail to satisfy both requirements of equity and Pareto optimality. In practice, this zero-sum framing manifests in concrete allocation decisions, for example, expanding access to advanced genetic testing pathways (offering high diagnostic yield but substantial upfront costs), funding additional specialist referral capacity (improving expert access but limited by workforce scalability), or deploying AI-based triage tools (lower per-patient cost and broader population reach for initial screening but requiring infrastructure and validation).^49^^,^^50^ The rise of AI in medicine has intensified this disparity, as AI development favors conditions with large patient cohorts where incremental algorithmic gains deliver broader societal impact. Cost-effectiveness analyses further reinforce this bias.

Delayed RD diagnoses also impose significant burdens, with lifetime care costs three to five times higher than with early intervention.^51^ However, RD’s heterogeneous pathophysiology and small patient populations demand highly tailored models, which are costly to build and lack scalability. Systemic barriers, including fragmented data systems, absent standardized RD frameworks, and prohibitive computational costs for small datasets, further discourage investment in decentralized, disease-specific AI solutions. These challenges sustain resource allocation imbalances, eroding diagnostic equity for patients with RD and exposing structural deficiencies in AI-driven healthcare governance.

#### Proposed strategy: Fostering transparent and equitable resource allocation

Decisions about funding and deploying AI interventions in RD care should balance ethical imperatives with practical realities. In the scenario above, multidisciplinary governance bodies could apply equity-weighted cost-utility analyses to compare options, for instance, evaluating how AI triage tools enhance referral efficiency and reduce diagnostic odyssey costs versus expanding genomic testing or specialist networks.^49^^,^^50^ Multidisciplinary governance bodies, comprising clinicians, ethicists, patient advocates, and policymakers, should guide resource allocation through rigorous cost-utility analyses that prioritize unmet needs. Along these lines, the European Organization for Rare Diseases, a non-governmental organization (NGO) and patient-driven alliance, has fostered public-private partnerships that have influenced EU RD research funding^52^; in the US, the National Organization for Rare Disorders has driven orphan drug development.^53^ Private philanthropies, including the Bill & Melinda Gates Foundation, also fund early-stage RD research and catalyze multi-sector partnerships.^54^ Transparent and publicly led prioritization, coupled with independent audits, enhances accountability, while allocation criteria and an equity lens ensure benefits reach all patient groups.

In addition, by treating equity as a foundational criterion rather than a compromise, healthcare systems can achieve a fair balance between economic efficiency and social justice, fostering environments in which both priorities can be achieved. RD research often yields breakthroughs that extend well beyond its initial scope. Take PCSK9 inhibitors: this class of drugs, now a cornerstone of preventive cardiology for high-risk patients, was developed from studies of familial hypercholesterolemia.^51^ This example demonstrates that equitable investment in RD research can drive innovations with broad therapeutic impact. By channeling resources into understudied conditions, healthcare systems not only address unmet needs but also catalyze advances that benefit the wider population.

### Medical ethics

Exacerbated by the marginalized majority paradox and global neglect of RD (Table 1), the integration of AI into RD care raises profound ethical questions that extend beyond technical and logistical hurdles, carrying significant implications for societal equity. Although certain conditions, such as amyotrophic lateral sclerosis, have secured robust support through high public awareness, vigorous patient advocacy, and even celebrity endorsements, these high-profile successes serve only to underscore the widespread neglect of the vast majority of RDs. Without deliberate efforts to distribute attention and resources more equitably, AI innovations may inadvertently reinforce the very inequities they have the power to alleviate.

The use of AI in RD management also shapes public confidence in medical innovation. Ethical discussions must consider how these technologies redefine the social contract around healthcare, including who gains access to new tools and how society values a range of health outcomes. Evaluations of long-term impact should be grounded in patient experience and real-world costs, for example, by assessing how policy shifts affect equity of care or how new treatment models alter total expenditure over a patient’s lifetime.

#### Proposed strategy: Addressing broader ethical challenges

Sustainable integration of AI in healthcare requires dynamic ethical frameworks that evolve alongside technological advances. Adaptive guidelines addressing consent, transparency, accountability, and fair distribution of benefits must be co-created with diverse stakeholders. These aspects should be considered in ethics review committees through applying equity frameworks, conducting regular audits of AI systems, and overseeing ongoing risk assessments. Tailored training programs for developers and end users will cultivate shared responsibility for ethical practice. However, a significant equity challenge remains unresolved. The regions that stand to benefit most from AI in healthcare, typically LMICs with constrained data infrastructure and limited workforce capacity, face substantial systemic barriers to implementation. To ensure that AI promotes dignity and equitable outcomes for all patients with RD, we must establish not only open data and knowledge repositories but also strengthen international cooperation mechanisms that ensure capacity and infrastructure development.

Importantly, positioning equity as a core ethical goal in RD AI requires that it be assessed through explicit, falsifiable criteria. Evaluation should prioritize whether performance disparities across clinically and socially meaningful groups are reduced and whether the lowest-performing or least-represented populations experience tangible gains. Equity-centered evaluation metrics should include (1) stratified and worst-group performance reporting,^55^ (2) disparity-aware error metrics,^56^ (3) counterfactual and robustness-based evaluations when subgroup sizes are small, and (4) longitudinal and deployment-level indicators of equity impact. Treating equity as a measurable outcome rather than a rhetorical commitment ensures that AI systems intended to support RD care can be meaningfully evaluated.

Notably, RD remains a predominantly high-income-country concern, as reflected by the geography of patient advocacy and NGO efforts, while LMIC priorities focus on infectious diseases of poverty.^57^ This neglect introduces an additional layer of structural discrimination, marginalizing individuals with RD in resource-constrained settings and exacerbating global health inequities. To bridge this gap, specific actions are needed: prioritizing LMIC representation and leadership in global AI governance forums; supporting locally led digital registries and networks (such as Brazil’s initiative for RD health indicators and care coordination^58^); investing in targeted capacity building, including training programs for local clinicians and researchers in AI tool deployment and ethical data curation; and developing and disseminating open-source, low-compute AI models optimized for resource-constrained settings. Complementary media outreach, patient organization partnerships, and professional society engagement can sustain public attention and drive fair resource allocation for RD care worldwide.

## Conclusion

Advancing equity in RD care demands a concerted, multidisciplinary effort to harness AI’s transformative potential while safeguarding ethical principles and patient rights. By prioritizing inclusive data collection, mitigating algorithmic biases, and strengthening privacy-preserving governance, stakeholders can develop robust, generalizable models that serve marginalized populations. Transparent resource-allocation frameworks and sustained investment in infrastructure, including LMIC-specific adaptations and partnerships, will ensure equitable access, especially in LMICs. Dynamic ethical guidelines, continuous fairness audits, and collaborative governance are essential to align innovation with social justice. This is not merely an aspiration for technological advancement; it represents a profound paradigm shift in how we approach care for often-overlooked diseases, transforming isolation into connection and diagnostic uncertainty into clarity. Ultimately, centering RD communities will catalyze AI-driven breakthroughs that empower all patients equitably, ensuring that no patient is left behind in the promise of precision medicine.

## Acknowledgments

We acknowledge funding from the 10.13039/501100012166National Key R&D Program of China (2024YFB4710200 and 2024YFB4710205); the 10.13039/501100001809National Natural Science Foundation of China (82388101 and 82271122); the 10.13039/501100003399Science and Technology Commission of Shanghai Municipality (20DZ2270800); the Shanghai Key Clinical Specialty, Shanghai Eye Disease Research Center (2022ZZ01003); the Shanghai Municipal Commission of Health and Family Planning Project (2022XD006); the Shanghai Jiao Tong University 2030 Initiative (WH510272301); the Shanghai Three-Year Plan for the Inheritance and Innovative Development of Traditional Chinese Medicine (2-5-1), Hainan Province Science and Technology Special Fund (ZDYF2024LCLH004); the Research to Prevent Blindness Career Development Award; and the Gills AI Innovation Center.

## Author contributions

Conceptualization, H.Z. and C.L.; writing – original draft, C.L., Y.Z., and H.Z.; writing – review & editing, C.L., Y.Z., C.A.L., J.S., B.X., T.Y.A.L., T.P., Z.W., K.D., K.J., H.Y., H.Y.T., F.K., N.Z., L.Z., and H.Z.; figure and table design, C.L., Y.Z., H.Y., and Z.W. The final version of the paper was critically reviewed and approved by all authors.

## Declaration of interests

The authors declare no competing interests.

## Declaration of generative AI and AI-assisted technologies in the writing process

During the preparation of this work, C.L. and Y.Z. used ChatGPT and Grok to improve the clarity, readability, and English grammar of the work. After using this tool, the authors reviewed and edited the content as needed and take full responsibility for the content of the publication.
